# Factors correlated with financial hardship among cancer patients during the COVID-19 pandemic

**DOI:** 10.1371/journal.pone.0342984

**Published:** 2026-03-09

**Authors:** Sara P. Myers, Carolyn Tsung, J. C. Chen, Yevgeniya Gokun, Jesse Plascak, Mohamed I. Elsaid, Ashley Rosko, Carolyn J. Presley, Electra D. Paskett, Ann Scheck McAlearney, Samilia Obeng-Gyasi

**Affiliations:** 1 Division of Surgical Oncology, Department of Surgery, The Ohio State University Wexner Medical Center and James Cancer Hospital, Columbus, Ohio, United States of America; 2 CATALYST, Center for the Advancement of Team Science, Analytics, and Systems Thinking in Health Services and Implementation Science Research, College of Medicine, The Ohio State University, Columbus, Ohio, United States of America; 3 Washington University in St. Louis, St Louis, Missouri, United States of America; 4 Center for Biostatistics, College of Medicine, The Ohio State University, Columbus, Ohio, United States of America; 5 Division of Cancer Prevention and Control, Department of Internal Medicine, College of Medicine, The Ohio State University, Columbus, Ohio, United States of America; 6 Department of Biomedical Informatics, College of Medicine, The Ohio State University, Columbus, Ohio, United States of America; 7 Division of Medical Oncology, Department of Internal Medicine, College of Medicine, The Ohio State University, Columbus, Ohio, United States of America; 8 Division of Hematology, Department of Internal Medicine, College of Medicine, The Ohio State University, Columbus, Ohio, United States of America; 9 Department of Family and Community Medicine, College of Medicine, The Ohio State University, Columbus, Ohio, United States of America; Emory University, UNITED STATES OF AMERICA

## Abstract

**Purpose:**

Financial hardship from cancer care is associated with poor patient outcomes. Economic disruption during the COVID-19 pandemic may have exacerbated patients’ financial concerns. We explore how social deprivation index (SDI), clinical, and treatment-related factors impacted financial hardship in this observational secondary analysis using data collected prospectively during implementation of financial hardship screening from our NCI-designated center during COVID-19.

**Methods:**

Adults aged ≥18 years undergoing active treatment for stage 0-IV breast or lung cancer and who completed a 5-point Likert response-item screening for financial difficulty between 11/2020 and 11/2021 were included. Generalized estimating equations assessed associations between quartiles of zip-code level SDI, a composite obtained using data from US Census and American Community Survey, and binary outcome of financial hardship adjusting for relevant covariates*.*

**Results:**

Of 2245 patients, 87% identified as White, 9% as Black, 2% as Asian. The majority of patients identified as non-Hispanic (99%). Median age was 62 years old (IQR 53–71).The majority were treated for breast cancer (79%). Significant financial hardship (Likert responses ≥2) was reported by 7%. Most were married, had managed-care insurance, resided in urban settings, and had early-stage cancers (all p < 0.001). Of those included, 83% received surgery, 52% received chemotherapy, and 65% received radiation. Compared to the lowest SDI quartile, penultimate and highest quartiles were associated with financial hardship (Q3 aOR 1.86; 95%CI 1.13–3.05); Q4 aOR 2.05; 95%CI 1.15–3.63). Younger age, Black race, comorbidities, radiation, and chemotherapy were also associated with greater financial hardship (p < 0.05).

**Conclusion:**

In this study, greater SDI, younger age, Black race, comorbidities, receipt of radiation and/or chemotherapy were associated with financial hardship. These factors may guide focused screening of vulnerable populations to assist with equitable access to resources that offset the economic effects of cancer care.

## Introduction

The cost of cancer care is estimated to reach upward of $240 billion by 2030. [1] Although scientific advancements have improved survival, [2] frequent use of multimodality treatment and need for maintenance therapies contribute to medical debt. Consequently, patients may experience *financial toxicity* (FT)*,* a multidimensional phenomenon wherein material hardship, psychological response to costly care, and (mal)adaptive behaviors result in a diminished quality of life and poor clinical outcomes. [3] In December 2019, the coronavirus disease 2019 (COVID-19) instigated profound economic disruption with unemployment rising to ~15% in April 2020, [4] thereby increasing the proportion of patients at-risk for FT. Moreover, expenses incurred from treatment of COVID-19 and its sequelae may have made low-earning, unemployed, or fiscally vulnerable cancer patients especially susceptible to financial duress [5].

Despite efforts to mitigate FT, existing strategies have been underutilized. [6] Ascertaining risk for financial hardship is a major barrier to implementing a structured program to track patient experiences, i.e., financial navigation, and to delivering appropriate resources. [7] Data shows that only 25% of National Comprehensive Cancer Network (NCCN) member institutions perform targeted FT screening, [8] suggesting that establishing a feasible method of identifying patients with financial difficulties may improve resource distribution. Among programs that do employ targeted financial screening, certain populations (e.g., Black and non-English speaking patients) less frequently complete screening. [9] Moreover, several challenges impede the use of metrics for financial hardship/toxicity in the clinical setting; namely, instruments may be lengthy or require repeated use leading to survey fatigue, the majority have been designed for research rather than clinical contexts with little guidance on meaningful thresholds that can be used to guide intervention or referral for financial assistance resources, and there are concerns over generalizability given that metrics have been validated in those with advanced disease or specific cancer subtypes. [10,11] Our previous work demonstrated feasibility of using a single question to assess for financial hardship and guide referral to financial assistance for new and existing patients treated at our National Cancer Institute (NCI)-designated comprehensive cancer center. [12] Although previous studies have highlighted risk factors for financial difficulty among cancer patients (e.g., younger age, lower household income, identifying as a member of a racial/ethnic minority group, etc.), [13] few of these have specifically considered these in the context of COVID-19 or among patients being systematically screened. In this secondary analysis of data collected in the aforementioned screening pilot, we explore how sociodemographics, including social deprivation index (SDI), clinical, and treatment-related factors impact financial hardship among cancer patients evaluated during the height of the COVID-19 pandemic.

## Methods

### Study setting and patient selection

**Fig 1:**
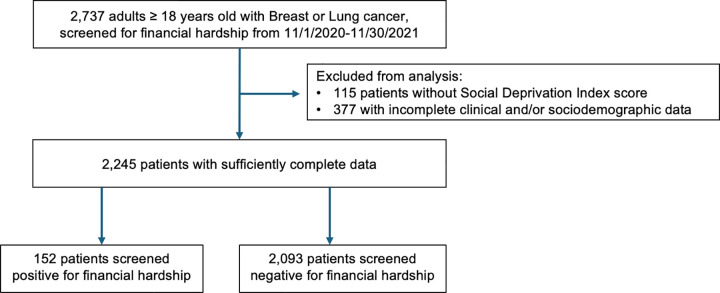
Study cohort.

Data were collected between November 1, 2020 and November 30, 2021 as part of feasibility testing of an electronic medical record (EMR)-based workflow to identify financial hardship at a single NCI-designated cancer center. Stakeholder input (i.e., clinicians, nursing staff, social workers, patient navigators, and financial counselors) informed a 3-step workflow described in the original manuscript: [7] 1) social determinants of health intake (including the screening item assessing financial hardship), 2) referral to social work for identified needs, and 3) assistance in securing need-based financial support. This study followed the Declaration of Helsinki and the Strengthening the Reporting of Observational Studies in Epidemiology (STROBE) guidelines [14] and was approved by The Ohio State University Office of Responsible Research Practices (2022C0154). With IRB approval and a waiver of consent, data recorded in the EMR as part of the aforementioned financial hardship screening was abstracted for patients aged ≥18 undergoing active treatment (systemic therapy, radiation, or surgery) for stage 0 through IV breast or lung cancer at The Ohio State University Comprehensive Cancer Center (Fig 1). A waiver of consent was granted because this minimal-risk secondary analysis used previously collected clinical data and recontacting patients would have been burdensome. Study size was pragmatic based on data acquisition that occurred between 9/20/2022-11/20/2022. Given the complexity and high costs of care associated with multiple cancers, history of previous cancer or solid organ transplant, patients with these diagnoses were excluded from analysis.

### Variables of interest

The primary outcome of interest for this analysis was financial hardship, which was assessed using a single item derived from the Study of Women’s Health Across the Nation study: “*How hard is it for you to pay for the very basics like food, housing, medical care, and heating?”* [15] Responses options were on a 5-point Likert scale (0 = not hard at all, 1 = …, 3 = , 4 = very hard). For the purposes of analysis and to accommodate skewed data, [16,17] the responses were dichotomized (i.e., yes = Likert responses ≥2, no = Likert responses 0–1).

The primary independent variable of interest was the zip code level SDI. Annual SDI values were estimated from 5-year American Community Survey data and linked to participants based on year associated with zipcode. A two-year gap existed between US Census and American Community Survey data and year at which zipcode data was recorded (i.e., zipcode recorded in 2021 was linked to SDI estimation based on 2019 data). We chose SDI as it is a zip code-level validated measure of general socioeconomic disadvantage and zip codes are commonly available through EHR without need of intensive street address geocoding. [11,18] While other metrics for social disadvantage, namely social vulnerability index (SVI), were considered, SDI was selected as the more suitable option given that SVI might introduce greater misclassification bias because it requires granular census tract data rather than zip code-level data. [19] Additionally, SDI measures area-level socioeconomic deprivation using indicators of material disadvantage whereas SVI assesses broader domains relevant to community vulnerability to disasters and public health emergencies. [19] Although COVID-19 did represent a public health emergency, socioeconomic recovery in response to this crisis was underway by 2022. [20] Moreover, the intent of this study was to examine how area-level socioeconomic disadvantage as a measure of material deprivation impacted financial hardship, thereby yielding SDI as the index of choice. Zip code-specific scores were created using weighted factor analysis of seven indicators: percent of the population with less than a high school diploma, percent with household income <100% of the federal poverty level, percent unemployed, percent housing units rented, percent housing with >1 person/room, percent housing with no vehicle, and percent housing with single adult parents. [21] Scores were ranked 1–100 nationally and linked to each patient’s zip code from most recent visit recorded in the EHR. Ranks were analyzed as quartiles (least = 1–25, mild = 26–50, moderate = 51–75, high = 76–100) with increased SDI scores indicating greater social deprivation. [1] As sensitivity analyses and tests for linearity, we used alternative categorization of SDI in three levels (low = 1–25, middle 26–75, high≥75) and as continuous with per 10-unit intervals.

Clinically relevant covariates for inclusion in adjusted analyses were selected *a priori* based on existing literature. [22,23] These included: sociodemographic factors such as age, self-identified race (White, African American/Black, Asian, other) and ethnicity (Hispanic versus non-Hispanic), marital status (married, single, or other), and primary insurance (private, Medicaid, Medicare, other), whether residence was in an urban or rural area, National Cancer Institute (NCI) Comorbidity Index calculated based on past medical history at time of visit during which financial hardship screening was performed, [24] and treatment details consisting of receipt of chemotherapy, receipt of radiation therapy, and receipt of surgery. Because SVI incorporates race and ethnicity as area-level structural attributes while our models include these characteristics at the individual level, using SDI avoids conflating group-level structural composition with person-level sociodemographic effects and thereby maintains conceptual consistency in our analytic approach.

Secondary outcomes of interest included COVID-19 diagnosis prior to reporting financial hardship. Those identified as American Indian, Alaskan Native, Pacific Islander, multiracial, or other were collapsed into the “Other” category due to small sample sizes. Although cancer type and stage were recorded, they were not included as distinct covariates in the adjusted analyses given that recommended treatments would have been dictated by tumor type and stage, and, therefore there was concern for conceptual collinearity between these factors and received treatment modalities.

### Statistical analysis

Patients with non-missing clinical and/or sociodemographic variables were included in analyses. Descriptive statistics were used to explore how sociodemographic, clinical, and treatment characteristics varied by SDI quartile using Wilcoxon Rank Sum test and Chi-square/Fisher’s exact tests for continuous and categorical variables, respectively. Generalized estimating equations (GEE) for binary outcome with exchangeable correlation structure and the logit link to obtain odds ratios were used to assess the relationship between SDI and financial hardship adjusting for predetermined covariates. This GEE approach was used to account for clustering within zip codes.

For the sensitivity analyses, multiple imputations by chained equations were used to account for missing values. [25] Missing patterns and auxiliary variables were added to the imputation model to increase power and ensure satisfaction of the missing at random (MAR) assumption. Ten imputed data sets were created using logistic regression-based imputation models for binary and ordinal variables, discriminant function for non-ordered variables, and regression-based projected mean matching for continuous variables. Imputation-corrected parameters and standard errors (SEs) were combined using Rubin’s rule. [26] Exploratory analyses were aimed at understanding whether COVID-19 diagnosis in the 2 years prior was associated with financial hardship. All statistical analyses were performed using SAS version 9.4 (SAS Institute; Cary, NC; www.sas.com) and statistical significance was defined as p-value<0.05.

## Results

Of the 2,737 patients completing screening, 2245 were included in this secondary analysis (Table 1). 1777 (79%) and 468 (21%) were evaluated for breast cancer and lung cancer, respectively. The median age of screened patients was 62 (IQR 53,71) years old. The majority of patients were White (1944/2245 (87%)), with 212 (9%) identifying as Black, and 54 (2%) identifying as Asian. Of the patients included, 19/2245 (1%) identified as Hispanic. Across SDI quartiles, there were significant differences between marital status, insurance, residence in an urban setting and stage (p < 0.001 for all). 1854 (83%) patients underwent surgical resection of their primary tumor, 1195 (53%) received chemotherapy, and 1465 (65%) received radiation. Financial hardship was indicated by 7% of patients (152/2245), with the remaining indicating that paying for essential expenses was either “not hard at all,” (1923/2245) or “not very hard” (170/2245). Among the 152 patients classified as experiencing financial difficulties, most (109) described paying for essential expenses as “somewhat hard,” 25 as “hard,” and 18 as “very hard.”

**Table 1:** Sociodemographic, Clinical, and Treatment Characteristics by Social Deprivation Index.

Variable	Total (n = 2245)	Least Deprived (n = 1049)	Mildly Deprived (n = 384)	Moderately Deprived (n = 517)	Highly Deprived (n = 295)	p
**Age**, Median [IQR]	62 [53, 71]	63 [53, 71]	61 [51, 68]	63 [54, 72]	63 [56, 69]	0.023
**Race**						<0.001
White	1944 (86.59%)	952 (90.75%)	344 (89.58%)	490 (94.78%)	158 (53.56%)	
African-American/Black	212 (9.44%)	44 (4.19%)	29 (7.55%)	17 (3.29%)	122 (41.36%)	
Asian	54 (2.41%)	41 (3.91%)	4 (1.04%)	4 (0.77%)	5 (1.69%)	
Other	35 (1.56%)	12 (1.14%)	7 (1.82%)	6 (1.16%)	10 (3.39%)	
**Hispanic Ethnicity**	19 (0.85%)	12 (1.15%)	4 (1.05%)	1 (0.2%)	2 (0.68%)	0.229
**Marital status**						<0.001
Married	1427 (63.56%)	727 (69.3%)	256 (66.67%)	329 (63.64%)	115 (38.98%)	
Single	360 (16.04%)	121 (11.53%)	54 (14.06%)	84 (16.25%)	101 (34.24%)	
Other	458 (20.4%)	201 (19.16%)	74 (19.27%)	104 (20.12%)	79 (26.78%)	
**Insurance**						<0.001
Managed Care	1080 (48.11%)	556 (53%)	217 (56.51%)	220 (42.55%)	87 (29.49%)	
Medicaid	188 (8.37%)	56 (5.34%)	19 (4.95%)	50 (9.67%)	63 (21.36%)	
Medicare	932 (41.51%)	422 (40.23%)	141 (36.72%)	238 (46.03%)	131 (44.41%)	
Other	45 (2%)	15 (1.43%)	7 (1.82%)	9 (1.74%)	14 (4.75%)	
**Residing in an Urban area**	1588 (72.64%)	888 (86.13%)	222 (59.36%)	214 (43.23%)	264 (92.31%)	<0.001
**NCI Comorbidity Index**						<0.001
0	1344 (59.87%)	666 (63.49%)	244 (63.54%)	296 (57.25%)	138 (46.78%)	
1	508 (22.63%)	222 (21.16%)	92 (23.96%)	123 (23.79%)	71 (24.07%)	
2	215 (9.58%)	93 (8.87%)	29 (7.55%)	55 (10.64%)	38 (12.88%)	
3+	178 (7.93%)	68 (6.48%)	19 (4.95%)	43 (8.32%)	48 (16.27%)	
**Patient Type**						0.608
New	544 (24.23%)	262 (24.98%)	83 (21.61%)	128 (24.76%)	71 (24.07%)	
Established	1701 (75.77%)	787 (75.02%)	301 (78.39%)	389 (75.24%)	224 (75.93%)	
**Cancer Type**						<0.001
Breast	1777 (79.15%)	872 (83.13%)	309 (80.47%)	393 (76.02%)	203 (68.81%)	
Lung	468 (20.85%)	177 (16.87%)	75 (19.53%)	124 (23.98%)	92 (31.19%)	
**Clinical Stage**						<0.001
0	47 (2.09%)	30 (2.86%)	4 (1.04%)	11 (2.13%)	2 (0.68%)	
1	1209 (53.85%)	607 (57.86%)	196 (51.04%)	273 (52.8%)	133 (45.08%)	
2	457 (20.36%)	191 (18.21%)	99 (25.78%)	98 (18.96%)	69 (23.39%)	
3	222 (9.89%)	102 (9.72%)	39 (10.16%)	54 (10.44%)	27 (9.15%)	
4	310 (13.81%)	119 (11.34%)	46 (11.98%)	81 (15.67%)	64 (21.69%)	
**Received Surgery**	1854 (82.58%)	899 (85.7%)	330 (85.94%)	415 (80.27%)	210 (71.19%)	<0.001
**Received Chemotherapy**	1195 (53.23%)	534 (50.91%)	222 (57.81%)	285 (55.13%)	154 (52.2%)	0.094
**Received Radiation**	1465 (65.26%)	671 (63.97%)	251 (65.36%)	348 (67.31%)	195 (66.1%)	0.609
**Screened Positive for Financial Hardship**	152 (6.77%)	39 (3.72%)	24 (6.25%)	43 (8.32%)	46 (15.59%)	<0.001

Differences were noted across SDI quartiles in the proportion of patients who self-identified as Black, resided in an urban setting, had higher NCI Comorbidity index, had Medicare/Medicaid coverage, had higher stage disease, and received surgery (all p-values <0.001; Table 1). Similar associations occurred between these factors and self-reported financial hardship (S1 File). On multivariable analysis, residence in the highest and second highest quartiles of the SDI were associated with financial hardship compared to the lowest quartile (SDI Q3 OR 1.86; 95% CI 1.13–3.05; SDI Q4 OR 2.05; 95% CI 1.15–3.63, respectively). Younger age, Black race, higher NCI Comorbidity Index, receipt of radiation, and receipt of chemotherapy were also associated with financial hardship. Undergoing surgical resection was associated with lower odds of financial hardship, while receipt of chemotherapy or radiation was associated with higher odds of financial hardship (Table 2). The significance and the direction of the association between financial hardship and SDI did not change with sensitivity analyses using alternate categorization of SDI (i.e., low, middle, high levels and per 10-unit intervals, S1 File) or imputation to accommodate for missing data (n = 2,622) in the entire dataset. Of note, after comparing cases with and without missing clinical data across covariates, missingness was systematically associated with age, comorbidity burden, and visit type. As patterns for missingness were fully explained by observed characteristics rather than a theoretical basis to suspect dependence on unmeasured outcomes, the MAR assumption was upheld as appropriate. In exploratory analyses, COVID-19 diagnosis (p = 0.463) was not associated with financial hardship.

**Table 2:** Multivariable analysis modeling the outcome of financial toxicity (n = 2245).

Independent Variables	OR (95% CI)
**Age**	0.97 (0.95-0.98)
**Race**	
White	Ref
African-American/Black	1.83 (1.14-2.94)
Asian	2.59 (0.73-9.12)
Other	1.53 (0.52-4.50)
**Health Insurance**	
Managed Care	Ref
Medicaid	4.95 (2.77-8.85)
Medicare	3.27 (1.91-5.60)
Other	3.86 (1.47-10.15)
NCI Comorbidity Index*	
0	Ref
1	1.99 (1.28-3.10)
2	2.79 (1.68-4.62)
3+	2.88 (1.68-4.93)
**Received surgery**	0.52 (0.34-0.81)
**Received radiation** ^†^	1.58 (1.00-2.49)
**Received chemotherapy** ^‡^	1.50 (1.00-2.25)
**Social Deprivation Index (in quartiles)**	
Quartile 1 (1–25%): least deprivation	Ref
Quartile 2 (26–50%): mild deprivation	1.64 (0.96-2.79)
Quartile 3 (51–75%): moderate deprivation	1.86 (1.13-3.05)
Quartile 4 (76–100%): high deprivation	2.05 (1.15-3.63)

*The NCI Comorbidity Index is inversely proportional to 10-year survival estimates.

^†^95% CI does not include 1 but is presented this way due to rounding. Associated p-value is 0.042.

^‡^95% CI does not include 1 but is presented this way due to rounding. Associated p-value is 0.049.

Abbreviations: NCI: National Cancer Institute; OR: Odds Ratio; 95% CI: Confidence Interval.

## Discussion

In this secondary analysis of data collected as part of a pilot study investigating the feasibility of standard of care screening for economic hardship at a single NCI-designated cancer center, greater levels of social deprivation (as measured by the SDI) were associated with financial difficulty. Patients who were younger, self-identified as Black, had comorbidities, and underwent radiation and/or chemotherapy also had higher odds of experiencing financial hardship. As these data were collected during the COVID-19 pandemic, the clinical and sociodemographic factors found to be associated with financial hardship may inform targeted screening and referral for financial assistance in periods of global health crisis.

An increasing body of literature suggests that geographic social determinants of health influence access to medical care, [27] a particularly salient issue during COVID-19 when disruptions to workflow and supply-chain shortages further compromised care delivery. As such, several tools have emerged to delineate the complex relationship between socioeconomic status, residential neighborhood, and health-related outcomes. [28] Using area-level socioeconomic deprivation indices that are calculated from publicly available data (e.g., US Census) can provide estimates of relevant socioenvironmental factors. [29] These metrics have been shown to accurately reflect the distress associated with medical debt, i.e., FT, after breast cancer treatment. [30] Despite their utility in exposing disparities in care that could motivate health policy reform, errors resulting from application of aggregate data to individual patients, i.e., ecological fallacy, [31] are inherent to the use of area-level deprivation models. [32] For this reason, in keeping with an individualized approach to providing high-value care, creating a feasible and valid screen for FT that utilizes patient-level data to guide referral for assistance programs and financial navigation is important. This study showed that SDI was a strong correlate of patient-level financial hardship and may be used to target screening towards patients with higher likelihood of experiencing financial difficulty.

At present, there is no standardized approach for financial hardship screening. The PRO-TECT cluster-randomized trial, which investigated the use of patient-reported outcome metrics (PROM) versus usual care to monitor for symptoms in metastatic cancer patients, used a single item from the EORTC QLQ-C30 [33] validated survey to screen for financial hardship in the PROM arm. [34] The study concluded that significantly fewer patients experienced FT at centers that implemented screening to guide referral for financial counseling compared to those that did not (30% vs 39%, p = 0.01). In another study conducted at the North Carolina Basnight Cancer Hospital, investigators explored the use of various metrics to assess for financial hardship. These included the COmprehensive Score for financial Toxicity (COST), [35] the InCharge Financial Distress/Financial Well-Being Scale, [36] and the NCCN Distress Thermometer. [37] Participants of the study preferred a composite tool (COST PLUS) that was comprised of the COST measure with additional questions supplemented from the InCharge and NCCN Distress thermometer. In the recent protocol from Wheeler et al. for the Lessening the Impact of Financial Toxicity (LIFT) [38] multisite single-arm trial of financial navigation to reduce FT among adult oncology patients, patients who scored ≤22 on the COST scale (i.e., high risk for FT) were offered structured financial navigation. While available data from these trials indicate that any of these metrics might be successful when used to guide referral for financial counseling and/or navigation, it is important to note that both the EORTC QLQ-C30 and the COST instrument were validated in patients with metastatic cancer and may not be generalizable to all cancer patients. As an example, the COST items may not be pertinent to adolescent and young adult patients who are at high risk for experiencing FT but are still in the process of transitioning to fiscal independence. [39] Many of the questions may be more relevant to patients who have already initiated their cancer treatment(s) rather than to screen for baseline risk for financial hardship at the time of a new cancer diagnosis. Additionally, assessment length may lead to survey fatigue for patients and compromise feasibility and efficacy of these tools. Previous studies have demonstrated that cancer patients feel financial screening is valuable and prefer that it is performed regularly during the course of their care. [40] Frequent screening, however, represents a competing demand on an already strained healthcare infrastructure. [41] The single item of this study benefits from brevity and non-specificity of FT domain which might better characterize general FT earlier in the survivorship experience.

While area-level deprivation metrics identify important socioenvironmental factors for financial risk, the impact of individual characteristics may be overlooked. In this study, younger age, Black race, and pre-existing comorbid conditions were associated with positive financial hardship screen. Although, this study did not compare risk factors for FT pre- versus during/post COVID-19, investigations analyzing pre-pandemic patient-level data have shown that these same characteristics were associated with FT [42] and may, in fact, have been associated with experiencing more severe shifts in financial wellbeing due to the economic consequences of the pandemic. [43] Studies investigating racial/ethnic disparities in financial hardship during COVID-19 have shown that racial and ethnic minorities are more likely than their White non-Hispanic counterparts to experience domains associated with financial hardship (i.e., food insecurity, lost income, etc.). [44] Importantly, these shifts may not have been reflected in the SDI given the two-year gap which existed between US Census and American Community Survey data and year at which zipcode data was recorded. [45] While studies describing precise trends in deprivation indices over time as a reflection of COVID-19 are lacking, there is data to suggest that areas with higher SDI were disproportionately affected by job loss and reduced community mobility. [46,47] Unfortunately, disparities in screening for financial hardship suggest that these same vulnerable demographics are less likely to be targeted for financial screening or have access to a dedicated financial navigation. [48] Furthermore, using SDI rather than a patient-reported tool may fail to capture those with mid-range household incomes who experience psychological distress or exhibit coping behaviors to offset cost. [49] As a multidimensional construct, degree of FT does not necessarily demonstrate a linear relationship with degree of medical debt; recent studies have acknowledged that patients can experience financial hardship in spite of the safety-net that insurance and household income may provide [50].

This study had several limitations worth noting. As this approach to screening for financial hardship was implemented at a comprehensive cancer center, our data may not be generalizable to resource-limited settings where challenges to screening may be more pronounced. Zipcodes are larger than census tracts or block groups, which could lead to less precise geographic targeting and more variable trends with individual-level factors such as FT. [51] However, zip codes are readily available from EMR and do not require the geocoding procedures needed for determining smaller areas. Our sample population, which was relatively homogenous with respect to race and ethnicity, may not be applicable to more heterogenous populations. This is an especially salient considerations as 85% of the patients in our study cohort were White and non-Hispanic and studies have shown that financial hardship and clinically relevant sequelase stemming from resource barriers were more pronounced among racial and ethnic minorities during the pandemic. [52] Alternative measures such as SVI, which more broadly reflect financial hardship and also incorporate factors such as race/ethnicity, may have yielded different findings. [53,54] Although adolescent and young adults are at high risk for experiencing financial hardship, they are not well represented in this sample. As timing of screening for financial hardship varied with respect to phase of treatment, we may have failed to capture patients who did not experience hardship until after receiving certain treatments (e.g., chemotherapy). Finally, given that this data was collected as part of a feasibility pilot, efficacy data to understand whether financial hardship improved as a result of referral for financial assistance has not yet matured.

## Conclusions

In this study, greater social deprivation was associated with increased odds of experiencing financial hardship. Additionally, patients who were younger, self-identified as Black, had more comorbidities, and underwent radiation or chemotherapy had higher odds of experiencing financial hardship. These clinical and sociodemographic factors may be used to focus screening for vulnerable populations to ensure equitable access to resources that offset the durable economic effects of cancer care.

## Supporting information

S1 File **S1 Table. Patient characteristics stratified by financial hardship status. S2 Table.** Association between Social Deprivation Index (SDI) and financial hardship (n=2,245).
(DOCX)
