# Supplementary material for: Factors correlated with financial hardship among cancer patients during the COVID-19 pandemic
Source: PLoS One. 2026 Mar 9;21(3):e0342984. doi: 10.1371/journal.pone.0342984 (PMC12970929; doi:10.1371/journal.pone.0342984)
Supplement: S1 File — S1 Table. Patient characteristics stratified by financial hardship status. S2 Table. Association between Social Deprivation Index (SDI) and financial hardship (n=2,245). (DOCX) [file pone.0342984.s001.docx]

**Supplemental Table 1.** Patient characteristics stratified by financial hardship status.

|  |  | **Financial Hardship** | |  |
| --- | --- | --- | --- | --- |
|  | **Total** (n=2245) | **Yes** (n=152) | **No** (n=2093) | **P-value** |
| **Age** |  |  |  | 0.065 |
| Median (IQR) | 62 (53, 71) | 61 (55, 67) | 63 (53, 71) |  |
|  |  |  |  |  |
| **Race**, n (%) |  |  |  | <.001 |
| White | 1944 (86.6%) | 105 (69.1%) | 1839 (87.9%) |  |
| African American/Black | 212 (9.4%) | 38 (25.0%) | 174 (8.3%) |  |
| Asian | 54 (2.4%) | 5 (3.3%) | 49 (2.3%) |  |
| Other | 35 (1.6%) | 4 (2.6%) | 31 (1.5%) |  |
|  |  |  |  |  |
| **Ethnicity**, n (%) |  |  |  | 1.000 |
| Non-Hispanic | 2211 (99.1%) | 151 (99.3%) | 2060 (99.1%) |  |
| Hispanic | 19 (0.9%) | 1 (0.7%) | 18 (0.9%) |  |
|  |  |  |  |  |
| **Marital status**, n (%) |  |  |  | <.001 |
| Married | 1427 (63.6%) | 57 (37.5%) | 1370 (65.5%) |  |
| Single | 360 (16.0%) | 52 (34.2%) | 308 (14.7%) |  |
| Other | 458 (20.4%) | 43 (28.3%) | 415 (19.8%) |  |
|  |  |  |  |  |
| **Insurance**, n (%) |  |  |  | <.001 |
| Managed Care | 1080 (48.1%) | 33 (21.7%) | 1047 (50.0%) |  |
| Medicaid | 188 (8.4%) | 43 (28.3%) | 145 (6.9%) |  |
| Medicare | 932 (41.5%) | 68 (44.7%) | 864 (41.3%) |  |
| Other | 45 (2.0%) | 8 (5.3%) | 37 (1.8%) |  |
|  |  |  |  |  |
| **Residing in an Urban area**, n (%) |  |  |  | 0.127 |
| Yes | 1588 (72.6%) | 114 (78.1%) | 1474 (72.3%) |  |
| No | 598 (27.4%) | 32 (21.9%) | 566 (27.7%) |  |
|  |  |  |  |  |
| **Charlson Comorbidity Index**, n (%) |  |  |  | <.001 |
| 0 | 1344 (59.9%) | 48 (31.6%) | 1296 (61.9%) |  |
| 1 | 508 (22.6%) | 44 (28.9%) | 464 (22.2%) |  |
| 2 | 215 (9.6%) | 31 (20.4%) | 184 (8.8%) |  |
| 3+ | 178 (7.9%) | 29 (19.1%) | 149 (7.1%) |  |
|  |  |  |  |  |
| **Patient Type**, n (%) |  |  |  | 0.974 |
| New | 544 (24.2%) | 37 (24.3%) | 507 (24.2%) |  |
| Established | 1701 (75.8%) | 115 (75.7%) | 1586 (75.8%) |  |
|  |  |  |  |  |
| **Cancer Type**, n (%) |  |  |  | <.001 |
| Breast | 1777 (79.2%) | 68 (44.7%) | 1709 (81.7%) |  |
| Lung | 468 (20.8%) | 84 (55.3%) | 384 (18.3%) |  |
|  |  |  |  |  |
| **Clinical Stage**, n (%) |  |  |  | <.001 |
| 0 | 47 (2.1%) | 1 (0.7%) | 46 (2.2%) |  |
| 1 | 1209 (53.9%) | 38 (25.0%) | 1171 (55.9%) |  |
| 2 | 457 (20.4%) | 29 (19.1%) | 428 (20.4%) |  |
| 3 | 222 (9.9%) | 30 (19.7%) | 192 (9.2%) |  |
| 4 | 310 (13.8%) | 54 (35.5%) | 256 (12.2%) |  |
|  |  |  |  |  |
| **Chemotherapy**, n (%) |  |  |  | <.001 |
| Yes | 1195 (53.2%) | 105 (69.1%) | 1090 (52.1%) |  |
| No | 1050 (46.8%) | 47 (30.9%) | 1003 (47.9%) |  |
|  |  |  |  |  |
| **Surgery**, n (%) |  |  |  | <.001 |
| Yes | 1854 (82.6%) | 89 (58.6%) | 1765 (84.3%) |  |
| No | 391 (17.4%) | 63 (41.4%) | 328 (15.7%) |  |
|  |  |  |  |  |
| **Radiation**, n (%) |  |  |  | 0.037 |
| Yes | 1465 (65.3%) | 111 (73.0%) | 1354 (64.7%) |  |
| No | 780 (34.7%) | 41 (27.0%) | 739 (35.3%) |  |
|  |  |  |  |  |
|  |  |  |  |  |
| **SDI quartile**, n (%) |  |  |  | <.001 |
| Least Deprived | 1049 (46.7%) | 39 (25.7%) | 1010 (48.3%) |  |
| Mildly Deprived | 384 (17.1%) | 24 (15.8%) | 360 (17.2%) |  |
| Moderately Deprived | 517 (23.0%) | 43 (28.3%) | 474 (22.6%) |  |
| Highly Deprived | 295 (13.1%) | 46 (30.3%) | 249 (11.9%) |  |

**Supplemental Table 2.** Association between Social Deprivation Index (SDI) and financial hardship (n=2,245).

|  | Crude OR (95% CI) | Adjusted OR (95% CI) |
| --- | --- | --- |
| SDI    10-unit increase | 1.24 (1.16-1.32) | 1.11 (1.04-1.18) |
| SDI (tertiles)    Low (1-25)    Middle (26-75)    High (≥76) | Ref  2.21 (1.38-3.54)  5.15 (3.06-8.67) | Ref  1.77 (1.12-2.79)  2.05 (1.16-3.64) |

*Adjusted for age, race, insurance, CCI, surgery, chemotherapy and radiation therapy.
